# Peer2Me – evaluation of a peer supported program for adolescent and young adult (AYA) cancer patients: study protocol of a randomised trial using a comprehensive cohort design

**DOI:** 10.1186/s12885-024-12547-5

**Published:** 2024-07-02

**Authors:** Hannah Brock, Sarah Dwinger, Corinna Bergelt, Annekathrin Sender, Kristina Geue, Anja Mehnert-Theuerkauf, Diana Richter

**Affiliations:** 1 Department of Medical Psychology and Medical Sociology, University Medical Center Leipzig, Comprehensive Cancer Center Central Germany (CCCG), Philipp-Rosenthal-Straße 55, 04103 Leipzig, Germany; 2 Department of Medical Psychology, University Medical Center Hamburg-Eppendorf, Martinistraße 52, 20246 Hamburg, Germany; 3 Department of Medical Psychology, University Medicine Greifswald, Walther-Rathenau-Straße 48, 17475 Greifswald, Germany; 4 Department of Psychosomatic Medicine and Psychotherapy, Medical Faculty, Otto-von-Guericke-University Magdeburg, Leipziger Str. 44, 39120 Magdeburg, Germany

**Keywords:** AYA, Cancer, Peer support, Mentoring, Emotional support, Young adults, Psychooncology

## Abstract

**Background:**

Developing cancer in young adulthood is a non-normative life event and associated with adverse physical, social and psychological consequences. High psychological distress is common in AYA cancer patients including anxiety, depression or fear of recurrence. At the same time, it is well known that AYA often report unmet needs for support, particularly in terms of informational exchange and emotional support from peers in order to benefit from shared experiences and enhance self-efficacy. Especially in the AYA group, interactions with other same-aged cancer patients may represent an essential resource in terms of coping with the disease, as family members and friends are often overwhelmed and struggling with helplessness. Currently, there is a lack of professional support services using peer support (e.g. psycho-oncological support, aftercare consultations, social legal counselling) or evaluated peer support interventions in Germany. Our aim is to assess the effectiveness of the Peer2Me intervention for AYAs, in which acute patients (mentees) are accompanied by an AYA survivor (mentor) over a period of three months.

**Methods:**

A prospective Comprehensive Cohort Design with repeated measures will be used to evaluate the effectiveness of Peer2Me for AYA. A sample of 180 patients in active cancer treatment aged 18 to 39 years will be enrolled and randomized to the intervention or control condition (a single AYA-specific consultation). Following mentor training, mentees and mentors are matched by diagnosis, age, and gender. The primary outcome is self-efficacy; secondary outcomes include measures of anxiety, depression, health literacy, life satisfaction and social support life. Outcomes will be measured at baseline before the intervention (t1), immediately after completion of the three-month intervention (t2) and three months after completion the intervention (t3). For the final analyses, we will use an intention-to-treat approach (ITT) and compare patients in the assigned treatment groups.

**Discussion:**

Peer2Me might be an important addition to existing professional psychosocial support services for young cancer patients. At the end of the study, a psycho-oncological intervention for young cancer patients undergoing acute treatment should be available, from which both mentors and mentees could benefit. The long-term continuity of Peer2Me should be ensured through collaboration with different partners.

**Trial Registration:**

The study was retrospectively registered on February 4, 2022 at clinicaltrials.gov (NCT05336318).

## Introduction

Cancer is a critical life event that can have a negative impact on quality of life, regardless of age. A cancer diagnosis during young adulthood presents individuals with unique challenges that must be navigated alongside typical developmental tasks, such as completing education or starting a family [1]. Stress induced by the disease and its treatment, including side effects, prolonged hospital stays resulting in social isolation, financial losses due to altered professional activities, or the loss of independence, can lead to significant psychological and social consequences, such as feelings of helplessness, anxiety, depression or familial conflicts [2].

AYAs are characterized by heightened vulnerability, particularly in terms of emotional distress. Research on the mental well-being of young adults with cancer indicates that up to 32% experience heightened psychological distress, anxiety, depression, and post-traumatic stress disorders [3–5]. Recent scientific research [6] indicates that AYA survivors are 55% more likely to report moderate distress compared to middle-aged or older cancer patients. The prospective longitudinal study, ’AYA-LE’, which included a sample of *n* = 514 young adults, confirms that 17% of patients have depressive symptoms and 42% exhibit anxious symptoms that remain stable over time [7]. Extended survival time results in approximately 60% of AYA patients experiencing high levels of fear of recurrence [8, 9], leading to reduced quality of life, both in the short and long term.

Self-efficacy is of critical importance in the well-being and outcomes of young cancer patients as it influences their ability to manage symptoms, distress, and the challenges associated with cancer treatment [10, 11]. Studies have indicated that self-efficacy influences cancer-related fatigue, health-related quality of life, and psychological adjustment in cancer survivors [12–14].

The impact of low self-efficacy on adolescent and young adult (AYA) cancer patients can have significant implications for their well-being and outcomes. Research indicates that low self-efficacy in AYA cancer patients is associated with higher levels of distress and poorer quality of life [15].

AYA patients with lower self-efficacy may encounter difficulties in coping with their diagnosis and treatment, leading to increased psychological distress and reduced overall quality of life [16, 17]. Additionally, low self-efficacy may impede AYA cancer patients’ ability to engage in self-care practices, adhere to treatment regimens, reduce healthcare utilization, and effectively cope with the challenges of cancer survivorship [16, 18].

These various strains are also evident in the reported care needs of AYAs. Research findings indicate that more than half of AYA patients have unmet information needs [19]. AYAs express a desire for information on long-term effects of cancer and treatments, including late effects, as well as financial matters and health-promoting behaviors related to sexuality, nutrition, and drug use [20, 21].

Sender et al. [22] demonstrated that patients’ psychological and healthcare information needs are consistent over time. If support needs remain unmet or only partially fulfilled, it may exacerbate psychological distress. Individuals experiencing psychological distress may require higher levels of support, making it necessary to implement a comprehensive psychosocial care plan to support AYAs throughout their treatment and survivorship [22].

Psycho-educational interventions and multimedia, such as web-based care-plans, are highly suitable for AYA [23, 24]. AYA cancer survivors, who are aware of available psychosocial services, such as cancer counselling centers, clinical liaison service, survivorship consultations, or social legal counseling, demonstrated a heightened motivation to use them during post-treatment care. Survivors must receive comprehensive information about psychosocial care to ensure positive outcomes [25].

Holland and colleagues identified several barriers to accessing psychosocial care. These barriers include personal factors such as severe physical impairment and lack of motivation, as well as systemic deficiencies such as a shortage of available services or insufficient interdisciplinary networking [26]. However, it is evident that regular and repeated screening and assessment of psychosocial issues and needs are absent. There is also a lack of widespread, age-appropriate support and psychosocial care specifically tailored to the needs of young cancer patients [27, 28].

Several studies have shown that the desire for peer-led support is highly significant for young cancer patients, who can derive substantial benefits from such interactions [29, 30]. Interaction with peers on an eye-to-eye level can help maintain normality and cope with upcoming developmental tasks such as identity formation or the development of a positive self-image [31, 32].

**Fig. 1:**
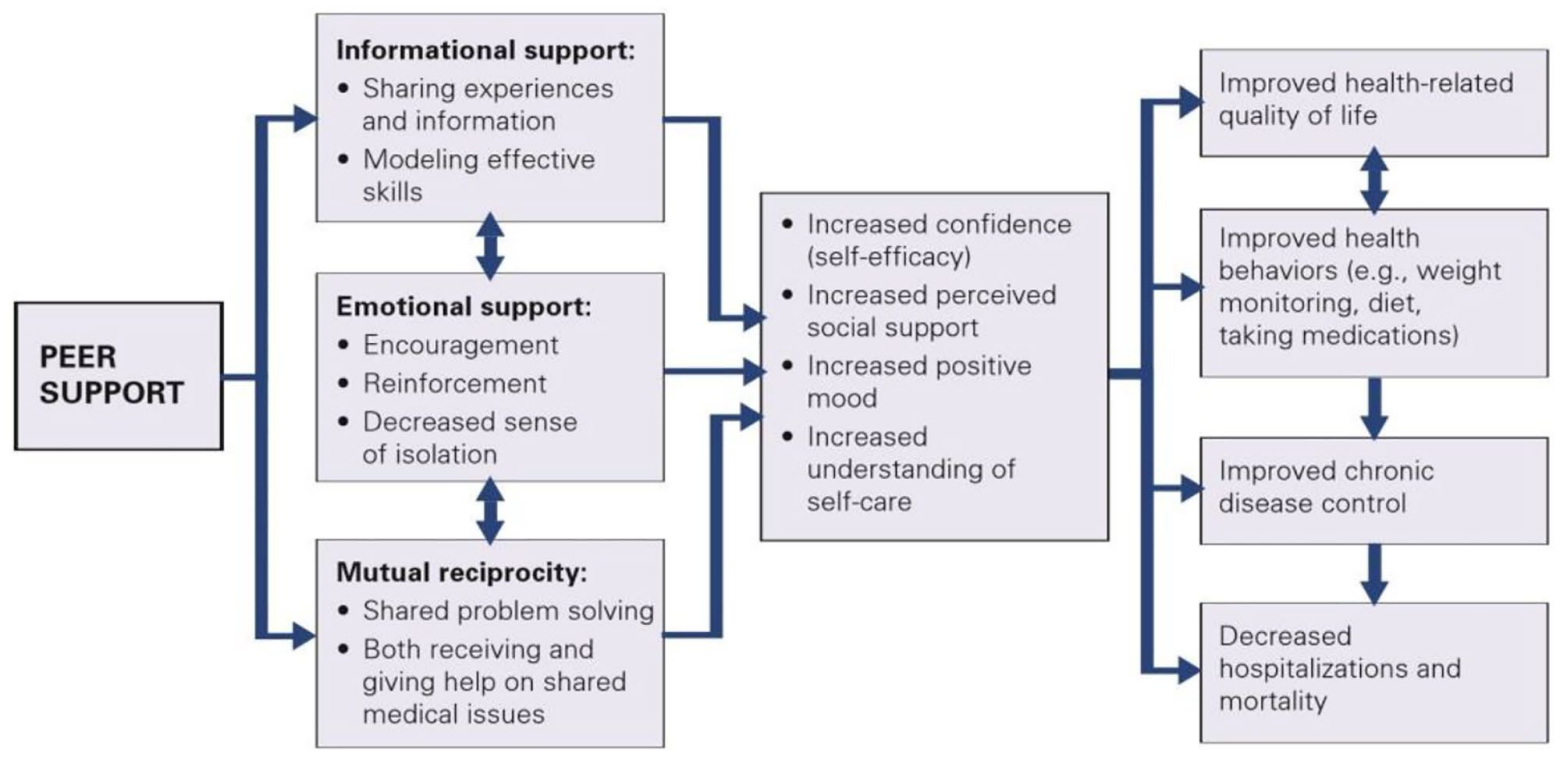
Hypothesized effects of peer support on self-care attitudes, behaviors and outcomes. (adapted from Heisler [38])

Cancer patients often do not want to burden their family and friends with their illness and are reluctant to communicate their fears and worries in this context [33, 34]. Several studies have confirmed that young cancer patients want to interact with others of the same age in order to rely on peer experiences and benefit from positive coping styles [35–37]. Heisler [38] emphasizes that the effectiveness of peer support is based on the non-hierarchical and reciprocal relationship between participants and has developed a model that clearly illustrates the benefits (Fig. 1).

The relationship between self-efficacy and peer support in young cancer patients is a significant factor in their coping mechanisms and overall well-being. Peer support has been found to play a critical role in how adolescents and young adults with cancer manage their diagnosis and treatment decisions, influencing their ability to cope effectively [39, 40].

Self-efficacy can be modified over time and through interventions aimed at alleviating symptoms associated with cancer therapy [10, 41, 42]. Peer support has been shown to have a positive impact on the self-efficacy of (AYA) cancer patients, as demonstrated in several studies [43–47].

The overall body of data from evidence-based peer support interventions for AYAs is very limited. The majority of studies have examined the effects of peer support in AYAs in group settings (e.g., professionally facilitated group intervention or internet-based groups), app-based or with healthy peers, and on psychosocial outcomes such as coping, quality of life, cancer-specific knowledge, social support or health-promoting behaviors [48]. Further research is needed on how to design peer-to-peer support programs as part of non-professional psychosocial support to provide the best care for young adult cancer with a particular focus on enhancing self-efficacy to improve coping behaviors.

## Methods

### Aim

The aim of the following study is to investigate how effective a mentoring program (Peer2Me) can be in increasing the self-efficacy of young cancer patients (mentees) in the short and medium term.

Hypothesis I: Participation in Peer2Me will lead to a significant increase in self-efficacy compared to the control group receiving standard psycho-oncological care. This effect will remain stable for at least 3 months.

In addition, the study aims to demonstrate whether Peer2Me is effective in reducing psychological distress and improving health literacy and social support.

Hypothesis II: Participation in Peer2Me will lead to a significant reduction in psychological distress compared to the control group receiving standard psycho-oncological care. These effects will remain stable for at least three months.

Hypothesis III: Participation in Peer2Me will lead to a significant improvement of health literacy compared to the control. This effect will remain stable for at least 3 months.

Hypothesis IV: Participation in Peer2Me will lead to a significant improvement of social support compared to the control. This effect will remain stable for at least 3 months.

Furthermore, we assume that the mentoring program can improve the mentors’ empathy skills and post-traumatic growth as well as their life satisfaction.

### Study design

The study is designed as a bi-center (university medical centers in Leipzig and Hamburg) comprehensive cohort study (CCD) [49] with a 1:1 allocation ratio over a period of 36 months and assessments at baseline and 3 and 6 months after the intervention. In this study design, eligible participants can choose to be randomized. In addition, patients who are not interested in participating in the Peer2Me intervention or the control group will be asked to complete the accompanying questionnaires at the three measurement time points. This will provide an additional no-intervention comparison group that will be relevant for comparison, in particular if the willingness to randomize in the study sample turns out to be very low.

### Recruitment

Acute AYA cancer patients (mentees) and mentors are recruited from January 2021 to June 2024 in the cooperating clinics (Hamburg and Leipzig in Germany) through personal approach by the study team. Eligible patients are invited to participate in a face-to-face meeting or by mail, and the invitations are followed up by telephone or e-mail. In addition, there is an ongoing successful cooperation with the psycho-oncological counselling services of the various cancer centers in Leipzig and Hamburg. Information about the study is also provided via study flyers and announcements as well as via social media postings (Facebook, Instagram), so that self-registration is also possible.

### Intervention

Peer2Me is a 1:1 mentoring program that aims to support acutely ill young adults with cancer with the help of a mentor in their immediate coping with the disease, thereby increasing their experience of self-efficacy and reducing their psychological distress. Between 2019 and 2020, a pilot study was conducted to evaluate the feasibility and utilization of this mentoring project [50].

The mentor’s support is intended to meet the need for emotional or psychosocial support. She or he assumes the role of a communication partner using her or his experience to help the mentee cope with the disease and the course of treatment.

Once mentors have been recruited, and if the results of the SCID and the initial interview are successful, they will attend mentor training. Participation in this training, which includes self-awareness components, is mandatory for mentors. The two-day training (6 h per day) takes place in a face-to-face or online setting in Hamburg or Leipzig with 10 to 12 participants and is conducted by psycho-oncologically experienced study staff. The content of the training includes basic client-centered communication skills and elements of motivational interviewing [51, 52]. This is intended to introduce the mentors to appropriate interview techniques such as active listening, open-ended questions, etc. In addition, the mentors are given the opportunity to learn about and reflect upon their role as a “former patient” and as a mentor. In this context, the boundaries of the mentors play an essential role. It is important that the mentors are protected in their role, are not pressured to act as medical experts or psychotherapists and are not expected to be able to answer expert questions. They should also be able to distance themselves from potentially excessive or inappropriate expectations of mentees. Other components include the acquisition of skills and exercises to improve disease management. In addition, mentors are provided with general medical (common tumor entities, long-term sequelae) and basic psychosocial knowledge about cancer in young adulthood and existing support services for the AYA patient group, such as support groups or cancer counseling centers. Both mentors and mentees also receive a folder with information (brochures, flyers, guides), and the latter will be able to contact their mentor, if they have any questions.

The intervention begins by matching the tandems (mentor and mentee) by age, diagnosis, and gender, if possible. At the first meeting of each tandem, a study staff member will be present to explain the Peer2Me study process and agenda, distribute cancer-specific and psychosocial information materials, and to conduct the baseline assessment (t1). Participants are either provided with a link to complete the standardized study questionnaire online using Lime Survey, or, if preferred, a printed version of the questionnaire is sent.

The tandem itself will then determine the frequency, duration, and type (face-to-face or telephone) of subsequent contacts between mentor and mentee over the following three months. However, at minimum of 4 documented contacts should take place for the purposes of the evaluation (fewer than 4 contacts are considered as a termination of intervention). The mentor documents each interaction, including the key issues discussed with the mentee, and informs the study team. After 3 months, a formal final meeting of the tandem will be held together with a member of the study team, during which the assessment t2 is conducted. The final survey (t3) will be sent to all mentees 3 months after the intervention.

Group supervision for the mentors will take place once a month. It will be provided by a psychologist with appropriate psycho-oncological and group therapy expertise.

### Control group

**Fig. 2:**
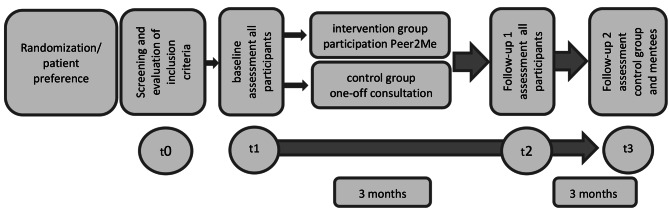
Study flow

Study participants in the control group will receive a one-time standardized 30-minute psycho-oncological consultation, including appropriate information material, as part of the AYA consultation at their study center. The t1 survey will also take place during this consultation. The t2 and t3 surveys will be conducted via e-mail or mail with appropriate invitations and reminders. A detailed study flow is shown in Fig. 2.

In the allocation or preference for the control condition, study participants receive a one-time standardized 30-minute counseling session with relevant information material in the AYA clinic at their study center. The t1 assessment also takes place during this AYA clinic counseling session. The t2 and t3 assessments will be conducted online or via postal mail with appropriate invitations or reminders.

### Eligibility criteria

#### Mentors

Study participants who act as mentors need to meet the following inclusion criteria: (1) a diagnosis of cancer at least 2 years before study inclusion; (2) current age and age at diagnosis between 18 and 39 years; (3) curative treatment approach; (4) ability to speak fluent German; (5) written informed consent to participate in this study; and (6) participation in a mentoring training. Patients will be excluded, if they are physically and mentally unable to participate in the study. To exclude mental disorders and suicidality, all mentors will be interviewed by a psychologist using the Structured Clinical Interview for DSM-5 Disorders - Clinician Version (SCID-5-CV) [53] before mentor training and the intervention (t0).

#### Mentees

Mentees are AYA patients undergoing acute treatment who receive emotional support and guidance from a mentor over a period of three months. All included mentees need to meet the following criteria: (1) diagnosis of cancer within the previous 6 months; (2) age between 18 and 39 years; (3) curative treatment approach; (4) ability to speak fluent German; and (5) written informed consent to participate in this study. Patients will be excluded, if they are physically and mentally unable to participate in the study and are undergoing psychotherapy. These criteria will be checked using electronic patient records and information provided by the treating oncologist.

### Randomization

The Comprehensive Cohort Design (CCD), or Patient Preference Trial, is an alternative to a randomized controlled trial. It is used when patient preference needs to be taken into account or when it is hypothesized that there is a relatively high preference for participation in a particular intervention [49, 54]. In the CCD, potential study participants are asked at the start of the trial if they agree to be randomized. If they agree, randomization takes place. If they refuse randomization, they can choose which study arm they will be assigned to. The CCD offers several advantages that are crucial for the successful implementation of this psycho-oncological intervention study, such as the possibility to include all potential study participants (including patients who refuse randomization) and a lower drop-out-rate in the control arm. If randomization is sufficient, an analysis similar to the RCT study is possible.

A limitation of the CCD is the difficulty in predicting the willingness of participants to be randomized. It is not possible to make clear statements about this in advance, especially as the present mentoring project is the first peer-supported intervention for young adult cancer patients in Germany.

If a sufficient number of study participants have been randomized in each study arm, analyses will be performed within the randomized arms and the non-randomized participants will be used to check external validity. If insufficient participants agree to randomization, all data will be analyzed as a prospective cohort study.

Potential study participants are fully informed about the study, the different study arms, and the main data to be collected. As a next step is to check the inclusion criteria and determine whether randomization can take place or whether there is a clear patient preference.

Randomization of study participants is performed within three working days by an independent documentation assistant of the Leipzig study center using the randomization software RITA. Block randomization with variable block length will be used to achieve a better balance. Stratification by gender and age will be performed for each study center to ensure structural equality for the intervention and control groups.

### Outcomes and measures

#### Primary outcome

Two standardized instruments are used to assess the extent to which the intervention contributes to an increase in self-efficacy (see Table 1). The hypothesis is that participants in the intervention group will report improved self-efficacy compared to participants in the control group (t2, t3), even three months after the intervention (t3).

#### General self-efficacy scale (GSE)

The validated German version of the General Self-Efficacy Scale is a self-administered instrument with 10 items for assessing a general sense of perceived self-efficacy with the aim to predict coping with daily hassles as well as to adapt various stressful life events [55]. It measures the expectation of subjective competence to act in the face of challenging situations such as cancer. A four-point Likert scale is used to determine the extent to which patients agree with the statements. The individual test score is calculated by summing up all items. Norm values of the German general population with an internal consistency of α = 0.92 and a sample of cancer patients with α > 0.90 are available [56, 57].

#### Cancer behavior inventory - brief version (CBI-B)

Self-efficacy in coping with cancer can be defined as a cancer patient’s confidence in his or her ability to develop adaptive coping behaviors. The German version of the short form of the Cancer Behavior Inventory (CBI-B-D) uses 14 items to describe coping behavior in the context of cancer [58]. Affected people estimate on a five-point Likert scale how confident they are in performing certain behaviors. By summing all 14 item-scores, a sum score is obtained, with higher scores indicating higher confidence in the ability to perform the coping behavior.

#### Secondary outcomes

As a secondary outcome, the data will provide information about the impact of the intervention on psychological distress (anxiety, depression), health literacy, life satisfaction, and social support on mentees (t1, t2, t3; see Table 1). The hypothesis is that participants in the intervention group will report less psychosocial distress, higher health literacy and life satisfaction and better social support compared to participants in the control group (t1, t2), even three months after the intervention (t3).

Similarly, for mentors, it is are expected that conclusions will be drawn on whether life satisfaction, empathy, and post-traumatic growth have changed after the 3 months of intervention (t1, t2).

#### Generalized anxiety scale (GAD-7)

The German version of the Generalized Anxiety Scale is a validated questionnaire that measures symptoms of generalized anxiety disorders and the symptom severity of generalized anxiety on a four-point Likert scale using seven items [59]. The individual item scores are summed to a total score, which can assume values between 0 and 21. The internal consistency in a representative German sample was α = 0.89 [60].

#### Patient health questionnaire (PHQ-9)

The Patient Health Questionnaire (depression module) measures depressive symptoms with nine items on a four-point Likert scale [61]. The PHQ-9 can be evaluated both categorically and by summing up the item characteristics. The scale sum value can reach values between 1 and 27.

#### European Health Literacy Survey Questionnaire (HLS-EU-Q16)

The HLS-EU-Q16 measures health literacy with 16 items and was developed from the long form of the HLS-EU-Q47 [62]. The items refer to various tasks and activities that are related to health care, disease prevention or health promotion. Respondents rate in each case how easy they think the corresponding task or activity is (“very easy,” “fairly easy,” “fairly difficult,” “very difficult”). A sum score from 0 to 16 can be calculated, and the latter can be classified as insufficient (< 9), problematic (9–12), and sufficient (13–16) health literacy.

#### Questionnaire of life satisfaction (FLZ-M)

The FLZ-M is a valid instrument that records the subjective assessment of satisfaction in various areas of life [63]. Life satisfaction is surveyed using the two modules “general life satisfaction” and “satisfaction with health”, each with eight items and an overall item. Subjective satisfaction and importance are assessed on a five-point Likert scale (0 = “dissatisfied” to 4 = “very satisfied”).

#### Berlin social support scales (BSSS)

The BSSS measure six dimensions of social support in a multidimensional approach: perceived social support, received social support, provided social support, need and search for social support, and protective cushioning in the sense of protecting others from stress [64]. Internal consistency was α = 0.8384 in a sample of *n* = 437 cancer patients.

#### Saarbrücken personality questionnaire on empathy (SPF)

The Saarbrücken Personality Questionnaire on Empathy is the German adaptation of the Interpersonal Reactivity Index [65, 66]. The SPF is a questionnaire for self-assessment of one’s own empathic abilities and consists of 16 items that measure both the cognitive and the emotional dimension of empathy on four subscales: imagination, empathic distress, empathic sympathy, and perspective taking. A five-point Likert scale (1 = “never” to 5 = “always”) is used to assess the extent to which the statements are true. Current norm values are available in age-graded tables.

#### Posttraumatic growth inventory (PTGI)

The Posttraumatic Growth Inventory (PTGI) measures the extent to which patients have experienced positive changes regarding cancer [67]. The questionnaire contains 21 items in five domains: new opportunities, relationship with others, personal strength, appreciation of life, and spiritual change. Using a five-point Likert scale, patients indicate the extent to which the item statements apply. Item scores are summed to a total score, with higher scores indicating higher posttraumatic growth. The PTGI is a reliable and valid instrument that has been translated into several languages.

**Table 1:** Study instruments

	Screening t0		Baseline t1		follow-up t2		follow-up t3	
instrument	mentor	IG/CG	mentor	IG/CG	mentor	IG/CG	mentor	IG/CG
sociodemographic	X	X						X
clinical variables	X	X						X
psychosocial variables	X	X			X	X		X
SCID-5-CV	X							
GSE				X		X		X
PTGI			X		X			
SPF			X		X			
CBI-B				X		X		X
GAD-7				X		X		X
PHQ-9				X		X		X
BSSS				X		X		X
FLZ-M			X	X	X	X		X
HLS-EU-Q16				X		X		X

Notes. IG = intervention group (mentees), CG = control group, SCID-5-CV = Structured Clinical Interview for DSM-5 Disorders Clinician Version, GSE = General Self-Efficacy Scale, PTGI = Posttraumatic Growth Inventory, SPF = Saarbrücken Personality Questionnaire on Empathy, CBI-B = Cancer Behavior Inventory - Brief Version, GAD-7 = Generalized Anxiety Scale, PHQ-9 = Patient Health Questionnaire Depression Module, BSSS = Berlin Social Support Scales, FLZ-M = Questionnaire of life satisfaction, HLS-EU-Q16 = European Health Literacy Survey Questionnaire

As part of the study, sociodemographic data, including age, partnership status, number of children, and employment status, were collected from all participants at t1. Additionally, clinical variables—such as diagnosis, time of diagnosis, and therapies—were recorded through self-report. Psychosocial variables encompassed the utilization of psychosocial support services and general inquiries into mental health, all of which were also documented at t1.

### Statistical analysis

#### Power and sample calculation

The number of cases was planned with the GPower program using a significance level of α = 0.05 and a power of (1-ß) = 0.8070. The primary outcome measure self-efficacy is used to demonstrate the effectiveness of the intervention. We make the following assumptions about changes in the intervention and control group: The pre-post effect size used is the effect size for self-efficacy.

d = 0.43, which could be found in the literature [68]. The expected controlled effect size requires a minimum of 90 subjects per group (180 subjects in total) measured with the U-test for independent groups. For this group size (*N* = 180), an analysis of variance with repeated measures (two groups, three measurements, correlation between measurements *r* = 0.5, epsilon = 1) can already demonstrate the following small effects: for comparisons between factors (IG vs. CG): f > 0.17; comparisons within factors (across measurement time points): f > 0. 09; and interaction effects between the two groups and time: f > 0.09. The sample thus has sufficient power to detect at least medium effects between the groups, as well as small effects within the groups and small interaction effects between the groups considering the measurement time points.

#### Statistical methods

Quantitative data will be analyzed using the statistical program SPSS 29.0 [69]. First, the items of the scales of the primary and secondary outcomes are checked for missing values. Items and complete sociodemographic variables that violate the MCAR (missing completely at random) assumption are identified in each subanalysis using Little’s MCARTest. Missing values are then estimated using the Markov Chain Monte Carlo (MCMC) procedure. This procedure yields reliable results even with high proportions of missing values for both the MCAR and MAR (missing at random) missing value patterns. On the other hand, it is suitable for any patterns of missing values, but also for monotonic patterns.

Descriptive analyses are calculated for all variables and presented as frequencies, means, standard deviations and ranges. Sociodemographic and medical predictors of intervention effectiveness are identified using stepwise backward regression. Non-dichotomous categorical characteristics are first subjected to multifactorial analysis of variance. Games-Howell correction or Bonferroni-Holm correction is used as a post hoc test, as appropriate. Correlation analysis is performed prior to each regression to avoid multicollinearity. Mean comparisons using one-way factor repeated measures analyses of covariance (ANCOVA) are used to test the hypotheses.

The first step is to compare only the randomized groups. In the next step, the preference groups are analyzed to determine differences in the effects compared to the randomization groups. Finally, all four groups are compared with each other, adding an indicator variable (randomization/preference) as a covariate. If the number of randomized study participants is too small, the data must be analyzed as a prospective cohort study.

## Discussion

Young adults with cancer are exposed to disease- and treatment-related stressors as well as various psychosocial stressors [27, 70]. Existing psycho-oncological research shows that AYA have unmet needs for information and support [71, 72]. Social support is crucial for this patient group, and in addition to family and friends, social interactions with peers play an important role [73, 74]. While social support from friends is very important for cancer patients of all ages, young adults with cancer may find it particularly difficult to share experiences and concerns with peers who are not affected by the illness in this age group. To date, there are only few evaluated peer-supported interventions in a one-to-one and face-to-face setting available to date that address the needs of young adults with cancer, either internationally and nationally [50, 75]. For this reason, the intervention was developed to promote interactions between young adult cancer patients that are beneficial to both groups of participants (mentors and mentees). Particularly in the acute phase of the illness, AYAs seek personal 1:1 contact with a young adult cancer survivor. Due to the shared experience in dealing with cancer and its effects and the non-hierarchical and reciprocal relationship between the participants, mentoring is an effective form of emotional and social support. At the end of the study, based on a proof of efficacy, an appropriate psycho-oncological intervention for young adult cancer patients undergoing acute treatment will have been developed. The long-term continuity of the program is planned to be ensured through collaboration with different partners.

## Data Availability

The datasets generated and analyzed during the current study are not publicly available due to required data protection regulations in patient information (which assures participants that the data are not passed on to third parties) but are available from the corresponding author upon reasonable request.
